# Systemic Amyloidosis After Liver Transplantation From a Donor With Hereditary Transthyretin Amyloidosis

**DOI:** 10.1016/j.jaccas.2026.110670

**Published:** 2026-10-21

**Authors:** Moises Abtibol Machado, Matheus Martins Monteiro, Denison Clark Corrêa de Miranda, Marcela Oliveira de Azevedo, Rodrigo Fernandes de Castro, Nayara do Carmo Santos Lima, Diogo Corrêa Lamberti, Andreza Araújo de Oliveira, Kátia do Nascimento Couceiro, João Marcos Bemfica Barbosa Ferreira

**Affiliations:** a Universidade do Estado do Amazonas (UEA), Manaus, Amazonas, Brazil; b Hospital Beneficência Portuguesa de São Paulo, São Paulo, São Paulo, Brazil; c Universidade NiltonLins, Manaus, Amazonas, Brazil

**Keywords:** cardiomyopathy, chronic heart failure, genetic disorders

## Abstract

**Background:**

Acquired transthyretin (TTR) amyloidosis after domino liver transplantation is rare and may manifest years after the procedure, with neurologic involvement.

**Case Summary:**

A 75-year-old man received a liver from a donor with TTR amyloid polyneuropathy. Twelve years later, he developed paresthesias, diarrhea, and blood pressure lability. Electroneuromyography showed a length-dependent, predominantly axonal sensorimotor polyneuropathy, with absent sympathetic skin responses, indicating autonomic involvement. Abdominal fat pad biopsy was Congo red positive, and TTR genetic testing was negative. Cardiac involvement was absent. He was treated with pregabalin and fludrocortisone, and patisiran was indicated as disease-modifying therapy.

**Discussion:**

The association of progressive neuropathy, dysautonomia, gastrointestinal manifestations, and domino transplantation favored acquired TTR amyloidosis over diabetic neuropathy. Reported cases show onset after 5-10 years, with neuropathy preceding cardiac disease.

**Take-Home Message:**

Domino transplant recipients require long-term follow-up, even with negative genetic testing and no initial cardiomyopathy, to enable early diagnosis and monitoring of systemic progression.

## Visual Summary

**Figure:**
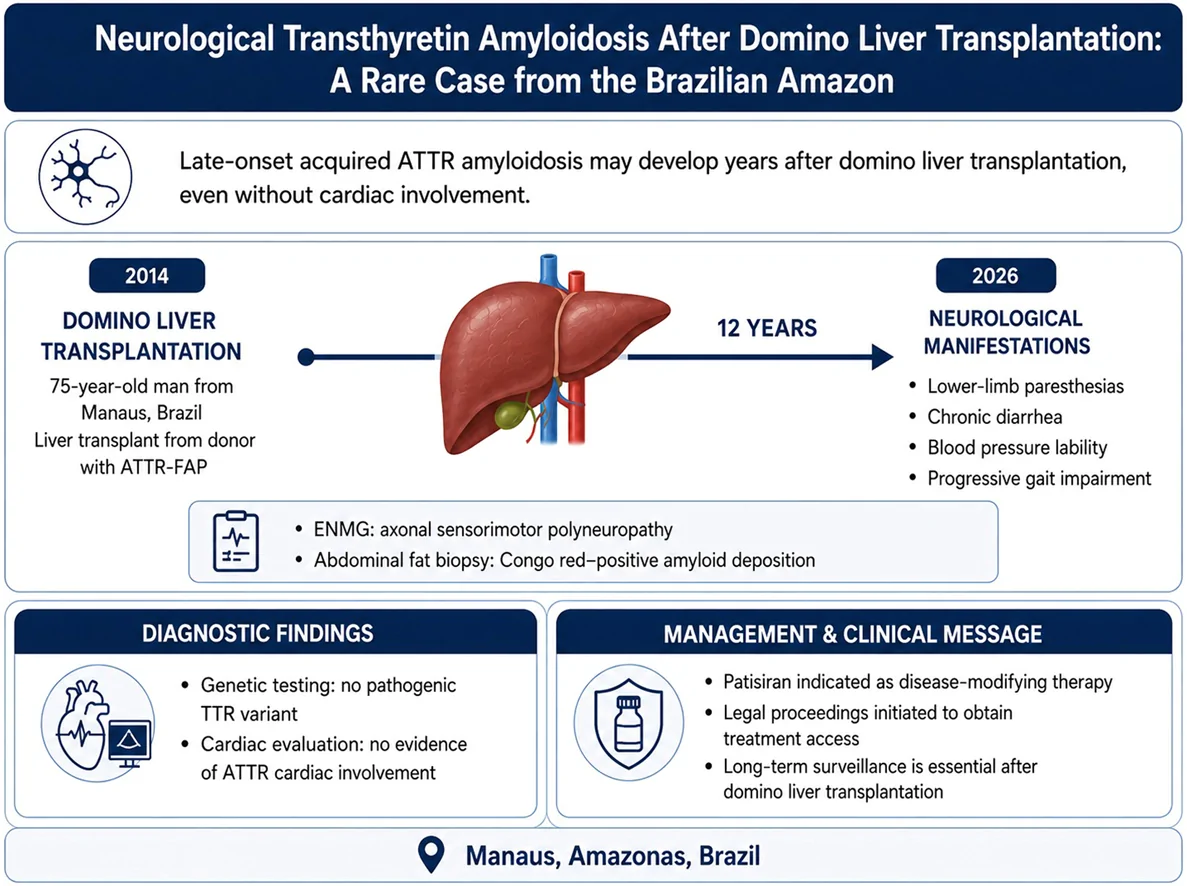

Hereditary transthyretin amyloidosis (ATTR) is a systemic disease characterized by the deposition of amyloid fibrils derived from mutant transthyretin (TTR), predominantly affecting the peripheral nervous system and the heart. The most common manifestations include progressive sensorimotor polyneuropathy, autonomic dysfunction, and infiltrative cardiomyopathy. Liver transplantation has historically been established as a classical treatment because it reduces the production of circulating mutant TTR.^1^^,^^2^

The clinical spectrum commonly includes progressive sensorimotor polyneuropathy, autonomic dysfunction, and infiltrative cardiomyopathy. Historically, liver transplantation has been considered cornerstone therapy because it reduces the production of circulating mutant TTR.^3^^,^^4^

## Past Medical History

A 75-year-old man, born in Manaus, Amazonas, Brazil, with a history of hepatitis C–related cirrhosis, underwent liver transplantation at the age of 64 years. The graft was obtained from a donor with a confirmed diagnosis of TTR familial amyloid polyneuropathy, characterizing a domino liver transplantation (DLT).

His medical history also included systemic arterial hypertension, diabetes mellitus, carpal tunnel syndrome, hypothyroidism, and nondialysis chronic kidney disease, with an estimated glomerular filtration rate of 29 mL/min/1.73 m^2^ and a serum creatinine level of 2.3 mg/dL in December 2025.

His regular medications included everolimus 0.5 mg every 12 hours, levothyroxine 88 μg once daily, dapagliflozin 10 mg once daily, losartan 50 mg every 12 hours, fludrocortisone once daily, pregabalin 75 mg every 12 hours, mirtazapine 15 mg at night, and atorvastatin 20 mg at night.

## History of Presentation

Twelve years after transplantation, the patient developed paresthesias in the lower limbs and plantar surfaces of the feet. The symptoms progressively worsened and were subsequently accompanied by chronic diarrhea and blood pressure lability, suggesting autonomic dysfunction.

During cardiological evaluation, he denied dyspnea, orthopnea, and paroxysmal nocturnal dyspnea. Physical examination revealed a regular cardiac rhythm, normal first and second heart sounds, no murmurs, and normal vesicular breath sounds without adventitious sounds.

Given the presence of diabetes mellitus, diabetic neuropathy was considered in the differential diagnosis. However, the clinical presentation with prominent autonomic dysfunction, chronic diarrhea, and rapid progression of symptoms, in addition to the presence of Congo red–positive amyloid deposition on abdominal fat biopsy, strongly supported the diagnosis of amyloid neuropathy rather than diabetic neuropathy.

## Differential Diagnosis

Given the presence of diabetes mellitus, diabetic polyneuropathy was initially considered. However, the prominent autonomic dysfunction, chronic diarrhea, blood pressure lability, and progressive neurologic symptoms were considered atypical for isolated diabetic neuropathy. The history of DLT from a donor with ATTR raised the possibility of acquired ATTR caused by the transplanted liver.

## Investigations

Electroneuromyography demonstrated a length-dependent axonal sensorimotor peripheral polyneuropathy, with small-fiber involvement and autonomic dysfunction in the lower limbs. An abdominal fat pad biopsy revealed Congo red–positive amyloid deposition, confirming systemic amyloidosis. Although mass spectrometry was not performed to further characterize the amyloid subtype, the clinical context of DLT from a donor with a TTR mutation makes acquired transthyretin amyloidosis (AqTTR) highly likely.^5^

Genetic testing for hereditary amyloidosis did not identify pathogenic variants in the TTR gene. In the context of the patient’s transplantation history, these findings supported the diagnosis of AqTTR following DLT.

The electrocardiogram showed sinus rhythm, low QRS complex voltage, a heart rate of 60 beats/min, and a normal electrical axis, and transthoracic echocardiography demonstrated a preserved left ventricular ejection fraction of 71%, normal ventricular chamber dimensions and wall thickness, and no echocardiographic findings suggestive of cardiac amyloid infiltration. Holter monitoring showed sinus rhythm, isolated atrial ectopic beats, and brief episodes of nonsustained supraventricular tachycardia.

Technetium-99m pyrophosphate myocardial scintigraphy demonstrated no cardiac uptake suggestive of TTR cardiac amyloidosis. Cardiac magnetic resonance imaging was not performed because of the risk associated with gadolinium administration in the setting of an estimated glomerular filtration rate below 30 mL/min/1.73 m^2^.

## Management (Medical/Interventions)

The patient continued symptomatic treatment for neuropathic pain and autonomic dysfunction with pregabalin and fludrocortisone, respectively. Given the progressive polyneuropathy associated with AqTTR, treatment with patisiran was requested as disease-modifying therapy.

Given the progression of TTR amyloid polyneuropathy, patisiran was indicated as disease-modifying therapy, and legal proceedings were initiated to secure access to the medication and enable treatment initiation.

## Outcome and Follow-Up

The clinical, histopathological, neurologic, and genetic findings were consistent with AqTTR following DLT, without evidence of cardiac involvement at the time of evaluation. The patient remains under multidisciplinary follow-up with the neurology and cardiology teams.

## Discussion

AqTTR following DLT represents a rare condition resulting from the continued production of mutant TTR by the transplanted graft. Although liver transplantation has historically been used as a disease-modifying therapy in patients with hereditary ATTR, the use of these livers in recipients without the disease may lead to the late development of systemic amyloid deposition. The prolonged latency between transplantation and symptom onset, often exceeding a decade, contributes to underdiagnosis and highlights the need for clinical surveillance in recipients with a history of DLT.^6^

In 2005, the first case of AqTTR was reported in a patient 8 years after DLT.^4^ A review of 16 cases of acquired familial amyloid polyneuropathy following DLT identified a male predominance (73%), a mean age of 60.7 ± 10.4 years, and Val30Met as the most frequent donor mutation (61.3%), with chronic hepatitis C as the leading underlying hepatic indication among recipients. De novo amyloid neuropathy manifested after a median of 7 years post-DLT (5.7 ± 3.2 years; range, 2 months to 10 years), with neuropathic pain and sensory loss as the most common initial symptoms, followed by erectile dysfunction, weakness, diarrhea, and orthostatic hypotension. Donor and recipient age were not associated with earlier onset of neuropathy.^7^

Individual cases corroborate this temporal pattern. A 68-year-old Portuguese patient with diabetes, transplanted for hepatitis C virus–related cirrhosis, presented 10 years post-transplant with thermal and painful sensory changes in both feet, accompanied by diarrhea and weight loss (10 kg over 1 year, 5 kg in the preceding 6 months); the diagnosis was established by salivary gland biopsy showing Congo red–positive amyloid deposition.^8^ In another case, in the United States, both donor and recipient developed a similar clinical phenotype—peripheral neuropathy, diarrhea, and weight loss—8 years after transplantation; the donor achieved remission for 4 years before progressing to death, whereas the recipient, who underwent retransplantation with a healthy liver, experienced disease recurrence after 5 years.^9^

A third case, involving a graft recipient with the Val30Met mutation, illustrates the value of differentiating this condition from diabetic neuropathy: At age 74 years, 8 years post-transplant, the patient developed numbness and paresthesia in the feet, progressing over 2 years to sensory involvement of the knees and hands and motor impairment of the ankles, with significant loss of mobility; renal dysfunction was also present, but glycated hemoglobin remained stable throughout, making diabetes unlikely as the sole cause of such a rapidly progressive and profound sensorimotor neuropathy. This patient was treated with patisiran (0.3 mg/kg every 3 weeks), later discontinued following a stroke, and died 4 years later from related complications.^10^

Finally, a series of 3 patients who underwent liver retransplantation showed that all developed neuropathic symptoms approximately 9 years after DLT; 2 of them, with advanced disease and marked autonomic and cardiac involvement at the time of retransplantation, continued to show progressive neurologic and systemic deterioration despite the procedure, whereas the third, retransplanted earlier and with a milder phenotype, achieved clinical stabilization over 2 years of follow-up.^11^

In recipients of DLT, neurologic manifestations typically precede cardiac involvement, most commonly presenting as progressive sensorimotor polyneuropathy and autonomic dysfunction. In the present case, the presence of paresthesias, gait instability, and autonomic symptoms, together with the absence of significant structural abnormalities on echocardiography, illustrates this predominantly neurologic presentation. The initial absence of infiltrative cardiomyopathy does not exclude the possibility of later cardiac involvement, supporting the need for longitudinal cardiologic follow-up even when structural imaging findings are normal.^12^

The diagnosis of AqTTR in recipients of DLT can be challenging when the recipient’s genetic testing is negative for TTR variants, potentially leading to delayed recognition. Continuous production of mutant TTR by the transplanted graft may result in systemic amyloid deposition even in the absence of a germline mutation, making clinical suspicion essential in the presence of progressive neuropathy and autonomic dysfunction. Therefore, a history of DLT combined with compatible clinical features and histopathological confirmation plays a crucial role in establishing the diagnosis.^13^

The differential diagnosis between amyloid neuropathy and diabetic neuropathy represents an important clinical challenge because both conditions may present with progressive sensorimotor impairment. In addition to the history of transplantation from a donor with a TTR mutation, the patient’s clinical context was also taken into consideration: insidious onset neuropathy developing after transplantation, with initial involvement of small fibers and autonomic dysfunction reported in the late phase of the disease, a pattern consistent with the temporal progression described for acquired amyloidosis following DLT.^8^ Furthermore, the patient had systemic involvement that could not be explained by diabetes alone, including renal and gastrointestinal involvement. Other findings made diabetic neuropathy less likely, particularly the disproportion between the severity of the neuropathy and the patient’s stable glycemic control. Electroneuromyography revealed a length-dependent axonal sensorimotor polyneuropathy, a finding compatible with both diagnoses. Additional testing showed that sympathetic skin responses (small C fibers) were absent in the lower limbs. Although this pattern of small-fiber involvement may also occur in diabetic neuropathy, its combination with the history of DLT, the clinical-metabolic disproportion, and multisystem involvement made acquired amyloid neuropathy the more likely diagnosis.^14^

The therapeutic management of these patients follows principles similar to those applied in hereditary ATTR, including the use of TTR stabilizers and gene-silencing therapies.^15^ Recent studies have shown that patisiran may lead to neurologic improvement in recipients of DLT who develop acquired amyloidosis, without increasing the risk of graft rejection or significant cardiovascular or renal adverse events, supporting the potential benefit of gene-silencing therapy in this setting.^16^

The unpredictable course of acquired amyloidosis following DLT highlights the need for multidisciplinary follow-up involving neurology, cardiology, and transplant teams. Clinical and imaging surveillance allows early detection of potential cardiac involvement and monitoring of neurologic progression, facilitating timely therapeutic adjustments. Furthermore, recognition of this clinical entity is essential to avoid diagnostic delays and optimize the longitudinal care of these patients.^17^

## Conclusions

This case demonstrates that AqTTR after liver transplantation may manifest late with predominantly neurologic features, even in the absence of cardiac involvement and with negative genetic testing in the recipient. Early recognition, histopathological confirmation, and multidisciplinary follow-up, together with disease-modifying therapy, are essential to limit disease progression and improve prognosis.

**Take-Home Message**

• Liver transplant recipients require long-term surveillance because acquired transthyretin amyloidosis may emerge years after transplantation and initially present with isolated neurologic manifestations.

**Visual Summary:**
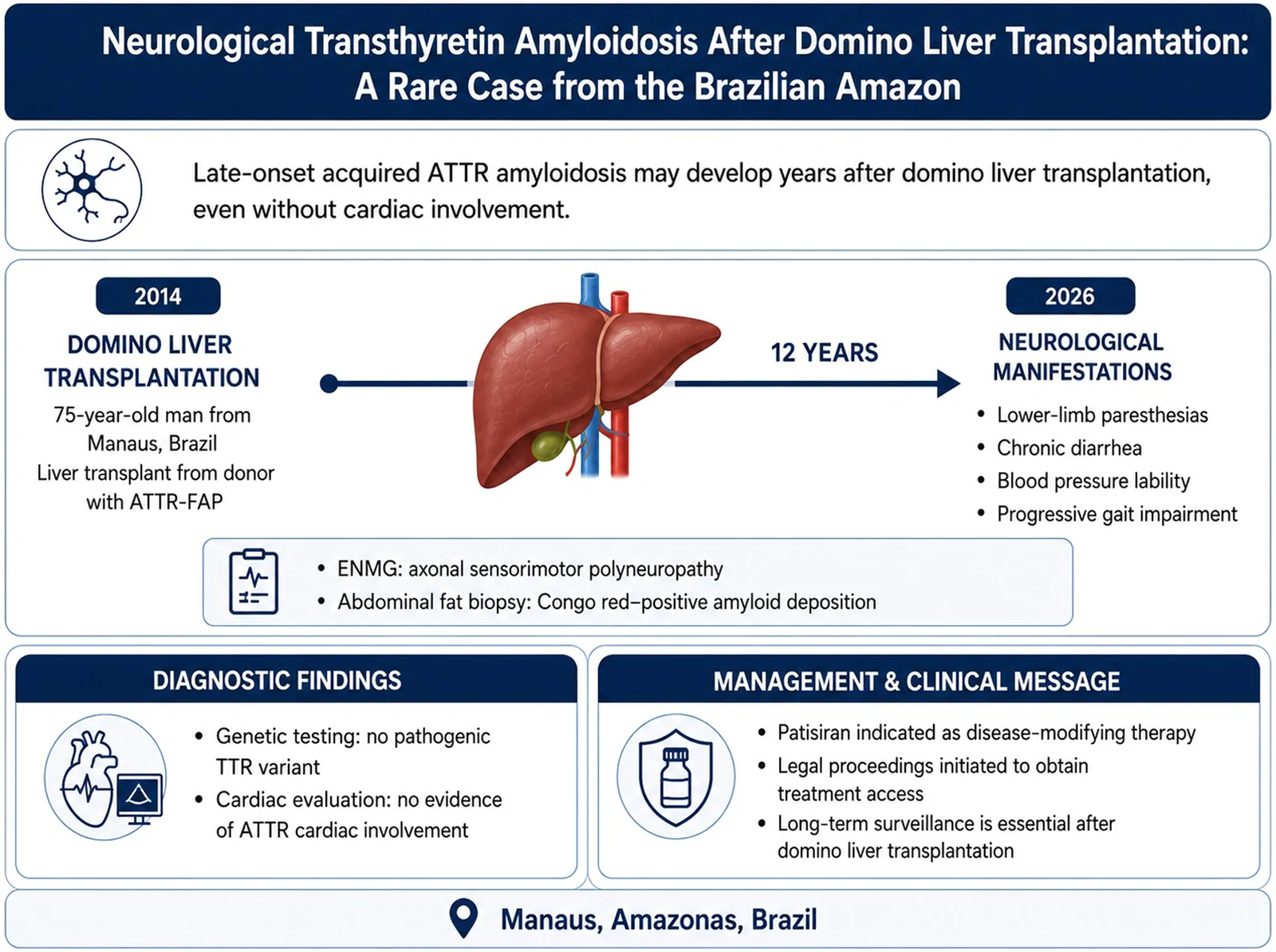
ATTR After Domino Liver Transplantation, Presenting With Progressive Neurologic and Autonomic Manifestations 12 Years After Transplantation, Without Cardiac Involvement ATTR = transthyretin amyloidosis; ATTR-FAP = transthyretin familial amyloid polyneuropathy; ENMG = electroneuromyography; TTR = transthyretin.

## Funding Support and Author Disclosures

The authors have reported that they have no relationships relevant to the contents of this paper to disclose.
